# Effectiveness of ketamine-assisted psychotherapy as a treatment for treatment-resistant depression: a systematic review

**DOI:** 10.1007/s00213-026-07003-0

**Published:** 2026-02-07

**Authors:** Rebecca J. Simpson, Mario F. Juruena

**Affiliations:** 1 Present Address: Centre for Affective Disorders-Department of Psychological Medicine Institute of Psychiatry, Psychology and Neuroscience-King’s College London, Room E3.17, PO72, 16 De Crespigny Park Denmark Hill, London, SE5 8AF UK; 2 South London and Maudsley NHS Foundation Trust Maudsley Advanced Treatment Service (MATS), London, UK

**Keywords:** Ketamine, Psychotherapy, Depression, Treatment resistant depression, Combine treatment

## Abstract

**Background:**

Major Depressive Disorder (MDD) is a common and debilitating condition. Current treatments fail to provide adequate relief in roughly one-third of individuals, resulting in treatment-resistant depression (TRD). Ketamine has emerged as a promising intervention for TRD, offering rapid and sustained antidepressant effects. However, benefits often require maintenance dosing, and long-term outcomes remain unclear. This has promoted interest in adjunctive approaches such as ketamine-assisted psychotherapy (KAP), which combines ketamine with structured psychological support.

**Methods:**

A comprehensive literature search was conducted via the OVID platform using Embase, Medline, Global Health, and APA PsycInfo. Keyword clusters related to “Ketamine,” “Diagnosis,” and “Psychotherapy” yielded 768 results. Screening followed PRISMA guidelines, resulting in 11 studies meeting inclusion criteria.

**Results:**

The selected studies clustered into two thematic categories: structured, protocol-based interventions and individualized, experiential approaches. Across all studies, KAP was associated with reductions in depressive symptoms, with some improvements sustained up to six months. However, among the three studies with control groups, no significant differences were found between KAP and control conditions. Methodological heterogeneity—including variability in treatment protocols, outcome measures, and study designs—limits the ability to draw firm conclusions or identify mechanisms driving KAP’s effects.

**Conclusion:**

KAP shows promise as a treatment for TRD, potentially offering meaningful and sustained symptom relief. However, limited evidence and methodological variability underscore the need for more rigorous research—particularly randomized controlled trials—to better understand its efficacy and mechanisms.

**Supplementary information:**

The online version contains supplementary material available at 10.1007/s00213-026-07003-0.

## Introduction

### Major depressive disorder

Major Depressive Disorder (MDD) is a common and debilitating disorder; approximately 5% of the global population experience symptoms (WHO, 2023). This figure steeply rises to around 16% for UK populations (ONS, 2022). MDD is characterised by feelings of depressed mood, or a loss of pleasure in things that previously brought joy. However, a plethora of other symptoms exist including weight, sleep or psychomotor changes; these make MDD inherently heterogeneous and difficult treat (APA, 2013). Accurately diagnosing MDD can be challenging due to symptom overlap with other psychiatric conditions, for example bipolar disorder (BD). Once a MDD diagnosis is confirmed, treatment options are mainly antidepressant drugs (ADD) or psychological talking therapies (NICE, 2022). Whilst other modalities exist, such as neuro-stimulation, these tend to be seen as a last-resort for those who are previously medication-resistant (Leiknes et al. 2012). Choosing the correct medication for clients can be challenging, and one must consider the individual requirements and unique symptom profile (Cleare et al. 2015). ADD can take up to 6 weeks to improve symptoms with sometimes modest outcomes, and there is on-going concern regarding the efficacy and safety of these drugs (Ioannidis 2008). Talking therapies, such as cognitive behavioural therapy (CBT) and behavioural activation (BA), can be used in tandem with ADD or as a stand-alone treatment (NICE, 2022). The best outcomes in treating MDD have been observed when using a combination of pharmacotherapy along with talking therapies (Cuijpers et al. 2014).

Despite these providing symptomatic relief in many patients, it is estimated that 1 in 3 of those with MDD have a treatment resistant form of the disorder (Phillips et al. 2023). Treatment resistant depression (TRD) is not universally defined, but the most common clinical definition is two or more failed treatments in current episode (Fekadu et al. 2018). Bringing this together, it is evident that new treatment options are necessary to help those suffering with the most complex form of the disorder.

### Ketamine

Ketamine is one such promising drug - initially discovered in the late 1960 s as an anaesthetic (Domino and Warner 2010) – which has been shown to possess antidepressant (AD) qualities at sub-anaesthetic doses (Berman et al. 2000; Zarate et al. 2006; McGirr et al. 2015; Marcatoni et al. 2020; Nikolin et al. 2023; Shen et al. 2024). The mechanism by which ketamine produces AD effects is not certainly known but is thought to be moderated by the N-methyl-D-aspartate receptor (NMDAR) in the brain (Kim et al. 2024). Regardless of the exact mechanism, there is robust evidence that a critical part of the AD effect of ketamine results from inducing a state of synaptic neuroplasticity (Li et al. 2010; Ly et al. 2018).

There is further discussion surrounding the classification of ketamine as a psychedelic. Psychedelics are defined as “substances that induce transient states of profoundly altered perception, thought and emotion” (Sessa 2005), and classification of a drug as a psychedelic is primarily based on their ability to induce such psychedelic state (Brys et al. 2023). It is widely accepted that classic psychedelics, like LSD, activate serotonergic 5-hydroxytryptamine receptor 2 A (5HT2A), causing a glutamate surge and a sustained increase in neuroplasticity (Gonzales-Maeso et al. 2007; Ly et al. 2018). Whereas dissociative anaesthetics, such as ketamine, produce similar psychedelic-like states primarily through antagonism of the NMDAR (Anis et al. 1983). More recently, it has been shown that ketamine’s acute AD effects, but not dissociative effects, require activation of the opioid system (Williams et al. 2018). This has led to further uncertainty regarding the classification of ketamine as a psychedelic. Whilst understanding this discussion within the field, ketamine will loosely be referred to as a psychedelic drug throughout the review.

### Ketamine as a treatment for TRD

Despite some uncertainty regarding the exact mechanism of action, there is a plethora of strong evidence highlighting the AD effects of ketamine within a depressed population. Systematic reviews and meta-analyses exist highlighting ketamine’s ability to not only alleviate depressive symptoms, but also improve suicidal thoughts (McGirr et al. 2015; Marcatoni et al. 2020; Nikolin et al. 2023; Shen et al. 2024). The AD effects appear to be rapid in onset - one study observed a reduction in depressive symptoms within 4 h (Daly et al. 2019), and continuation of ketamine treatment (in the form of repeated doses) has been shown to prevent relapse (Daly et al. 2019; McMullen et al. 2021). This evidence highlights the ability of ketamine to produce a rapid and prolonged AD effect. However, if stopped, the positive effects gradually disappear, highlighting the transient nature of the drug (Marcantoni et al., 2020). Indeed, the time-to-relapse following cessation for people with TRD is typically 2–4 weeks (McMullen et al. 2021).

### Long-term impact of ketamine treatment

As maintenance doses are required to sustain the AD effect of ketamine, it is worth examining the impact that prolonged use of the drug may have. There is evidence highlighting the negative effects that sustained ketamine use may have on the urinary tract system, specifically ulcerative cystitis as well as general urinary tract dysfunction (Li et al. 2011). As well as this, some concerns exist surrounding ketamine use and the effect this may have on the central nervous system - one study found that frequent ketamine use is associated with neurocognitive impairment and addiction (Morgan et al. 2010). However, some recent articles have found the opposite. A comprehensive review of ketamine’s addictive qualities concluded that it has a relatively low addictive risk when given with strict monitoring and judicious dosing (Ingrosso et al. 2024), and cognition is not impaired following both short- and long-term Esketamine treatment (Morrison et al. 2024). One systematic review examined the structural and functional brain changes associated with prolonged recreational use (Strous et al. 2022). It was found that long-term ketamine abusers, compared to controls, displayed: lower grey and white matter, abnormal frontal network organisation, and lower activity in brain regions for spatial memory and motor execution (Strous et al. 2022). This highlights the extent of damage that can be done to the brain if ketamine is abused. Interestingly, a possible dose dependent relationship was observed, as many of the brain changes were correlated with the amount and duration of ketamine consumption (Strous et al. 2022). Subjects in this review used at least 0.2 g ketamine twice weekly, which is 2–4 times the recommended clinical dosage (Strous et al. 2022), and so one must proceed with caution when extrapolating these results into a clinical setting.

As the use of ketamine within a psychiatric setting is relatively new, there is a lack of studies examining the long-term effect of therapeutic doses. There is some evidence suggesting an opposite effect of high and low doses of ketamine on the brain. One study found reduced connectivity after use of high doses (Chesters et al. 2016); lower dose ketamine was associated with an enhanced brain connectivity (Abdallah et al. 2017). Finally, it is worth noting that Esketamine, the enantiomer of ketamine, is used much more in clinical settings and appears to be less neurotoxic than racemic ketamine (Wajs et al. 2020). The type of molecule, dose and frequency of use appears to be important when considering the long-term side effect profile. There is a need for more studies examining the long-term effects of clinical standard ketamine.

### Ketamine-assisted psychotherapy

Ketamine-assisted psychotherapy (KAP) is a new treatment that offers the potential of improving and/or sustaining ketamine monotherapy. Combining psychotherapy with antidepressants is known to be more effective for those living with depression than either treatment modality alone (Cuijpers et al. 2014; NICE, 2022). Utilising this, there is emerging evidence suggesting that combining ketamine with psychotherapy could indeed improve outcomes. KAP is variably defined, and two loose groups seem to emerge - one aiming to sustain the AD effects of ketamine with psychotherapy following ketamine treatment, the other embeds talking therapy within the same session as ketamine. Throughout this review, KAP is used as an umbrella term describing intervention(s) and the first group will be referred to as the prescribed group, the second as the intuitive group. These groups have not been previously established in the literature and is an original idea put forward by the author. The prescribed group operates under the rationale that ketamine appears to induce a state of neuroplasticity (Li et al. 2010; Ly et al. 2018; Wu et al. 2021; Kopelman et al. 2023), providing an opportune window for psychotherapy to challenge some of the cognitive inflexibility seen in those with TRD (Jett et al. 2015; Wilkinson et al. 2017, 2019a, b, 2021). The intuitive group uses, what once was described as an undesirable side effect of ketamine, dissociation and the ‘emergence phenomenon’. When ketamine was initially being tested in the 1960 s (Domino and Warner 2010), participants experienced dissociative and hallucinatory experiences as the drug was metabolised, and since, there has been an effort to minimise such effects. However, there is emerging evidence that psychedelic experiences, including dissociation, may indeed correlate with positive outcomes (Sos et al. 2013; Luckenbaugh et al. 2014; Niciu et al. 2018; Ballard and Zarate 2020; Ko et al. 2022; Wolfson and Vaid 2024). These findings parallel the relationship between psychedelic experience and success of psilocybin (Griffiths et al. 2011), leading to the theory that a profound mystical experience, regardless of the drug inducing it, could improve TRD (Ko et al. 2022).

To conclude, TRD is a common and debilitating condition that can be treated with repeated ketamine therapy. Maintenance doses are needed, and the long-term impact of this remains largely unknown. KAP is a novel treatment option that offers the potential of sustaining and/or improving ketamine treatment alone. The aim of this paper is to systematically review evidence existing on the success of KAP in treating TRD. To the authors knowledge, one other review exists that examines KAP for TRD. However, this was published 2 years ago and was brief, reviewing 2 studies (Forcen and Nyer 2022). Other reviews examine the literature on the mechanism of action of KAP (Joneborg et al. 2022), or the literature regarding KAP as a treatment modality for a variety of disorders (Drozdz et al. 2022).

## Methods

Multiple databases (Embase, Medline, Global Health and APA PsychInfo) were searched via the online search tool OVID to gather relevant studies for the research question. The following search was conducted: (Ketamine or Esketamine) and (Depression or Major Depressive Disorder or Unipolar depression or Bipolar Disorder or Bipolar Depression or Bipolar I or Bipolar II or Treatment Resistant Depression or TRD or Difficult to Treat Depression or DTD) and (Psychotherapy or Ketamine Assisted Psychotherapy). The relevant clusters with keywords can be seen in Table 1.

**Table 1:** Clusters and keywords used for database search

Cluster	Keywords
Ketamine	“Ketamine” OR “Esketamine”
Diagnosis	“Depression” OR “Major Depressive Disorder” OR “Unipolar Depression” OR “Bipolar Disorder” OR “Bipolar Depression” OR “Bipolar I” OR “Bipolar II” OR “Treatment Resistant Depression” OR “TR-D” OR “Difficult to Treat Depression” OR “DTD”
Psychotherapy	“Psychotherapy” OR “Ketamine Assisted Psychotherapy”

**Fig. 1:**
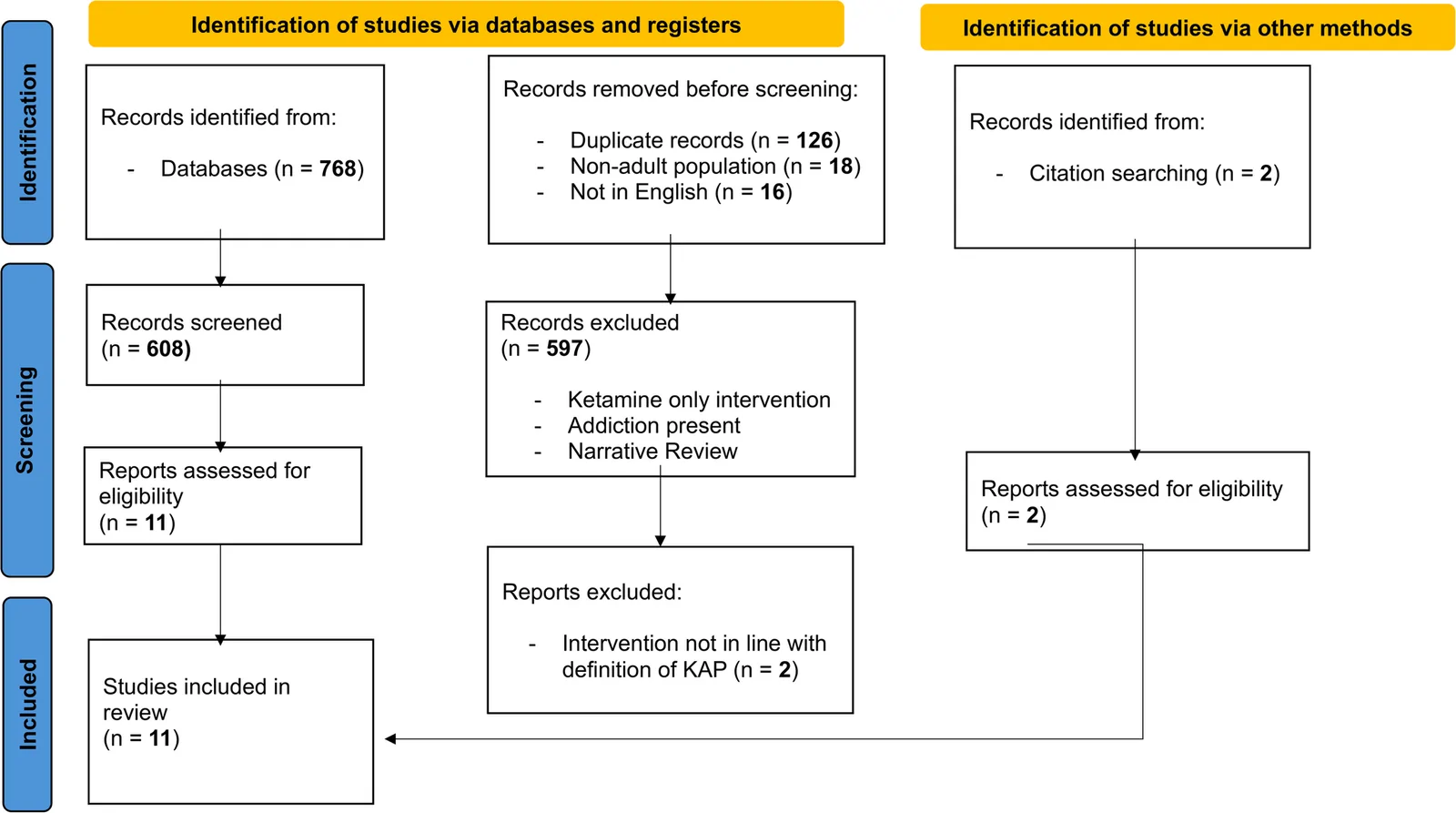
PRISMA flow chart showing the screening and selection process from the second OVID search (Page et al. 2021)

Using OVID, duplicate articles were removed and the filters “all adults” and “English language” were applied. Following this, using the online reference management programme Rayyan, the titles and abstracts were screened for eligibility. The search strategy is outlined in the Preferred Reporting Items of Systematic Reviews and Meta-Analysis (PRISMA) flow chart (Fig. 1) (Page et al. 2021). In addition to initial screening, the reference lists of included studies and relevant reviews were manually searched to find any additional articles.

The inclusion criteria of this systematic review were formulated utilising the PICO framework (i.e., population (P); intervention (I); comparison exposure (C), and outcome (O); (Frandsen et al. 2020).

### Inclusion criteria

 Participants:

- Adults $\:\ge\:18\:$with a diagnosis of MDD or Bipolar Depression (type I or II).
- Co-morbid diagnoses (e.g. PTSD and secondary addiction).

Studies:

- Observational or experimental (quantitative) study design (e.g., randomised control trials, non-randomised control trials, cohort studies, case-control studies, retrospective data analyses).
- Studies must include valid measures for depressive symptoms (e.g. PHQ-9).
- KAP (ketamine AND psychotherapy) alone OR against control group (e.g. KAP vs. ketamine alone).

#### Exclusion criteria

Participants:

- Aged * <18*.
- Primary addiction or substance misuse.

Studies:

- Animal studies.
- Those in languages other than English.
- Conference abstracts.
- Systematic reviews and meta-analyses.

As outlined in Fig. 1, of the 608 studies, 11 were identified from the database search and an additional two were identified from citation lists. The majority of papers were excluded due to: a ketamine-only intervention, primary substance misuse within a large proportion of the sample population, or the papers were narrative syntheses. If there was any uncertainty regarding the inclusion/exclusion of a study, a discussion took place with Dr MJ (co-author) until an agreement was made. This was relevant for two studies, as it was decided that the intervention did not align with the definition of KAP. These were removed from any further analysis. The search was initially conducted in October 2023 and was performed again at the end of October 2024 to ensure it was as up to date as possible (see supplementary material for original PRISMA chart, Figure S1) - there were no additional studies to include in this review. The review is registered on Prospero (CRD42024529443) and will be updated upon publication.

After selection, each paper was fully analysed and the following data was extracted: study characteristics (including sample size, demographic and main diagnosis of participants), study design (including when ketamine was given in relation to participants receiving psychotherapy), route and dose of ketamine, type and length of psychotherapy and main findings. The aim of this paper is to systematically review the literature on KAP and go on to make conclusions regarding its effectiveness in alleviating depressive symptoms. Due to the heterogeneous study design of the papers included, it was decided that a narrative synthesis with additional tables and graphs highlighting key results would be most appropriate. Where possible, reporting standards of included studies will be assessed with the Consolidating Standards of Reporting Trials (CONSORT) checklist (Schulz et al. 2010).

## Results

**Fig. 2:**
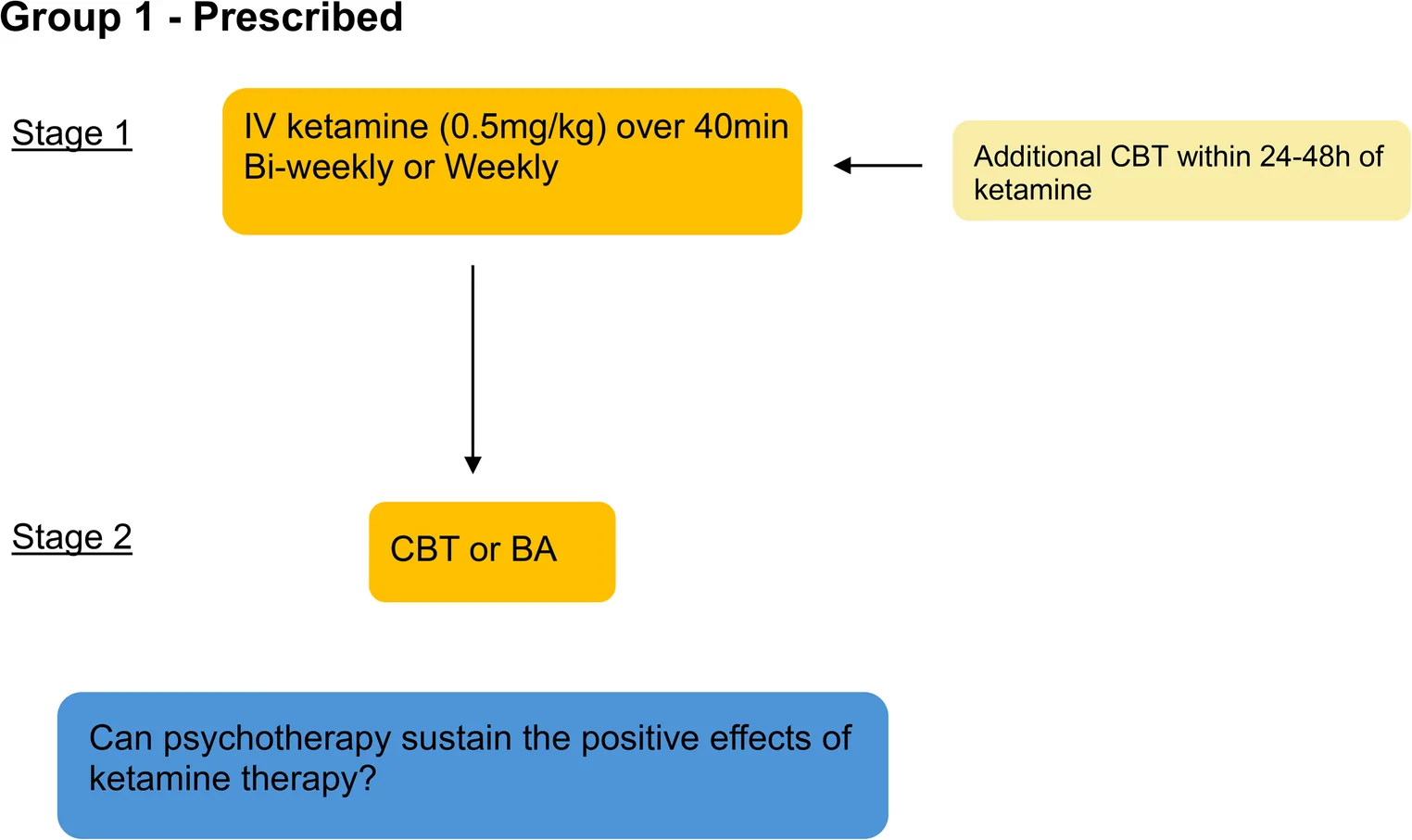
Flow diagram highlighting the main stages of studies in group 1. This group delivers ketamine therapy separately to psychotherapy, aiming to prolong the AD effects of ketamine. One study delivers CBT within 24–48 h of ketamine infusions in the first stage. Dark Yellow - Treatment Type; Light Yellow – Additional treatment; Blue – Study Aim. Abbreviations: Intravenous (IV); Cognitive Behavioural Therapy (CBT); Behavioural Activation (BA)

**Fig. 3:**
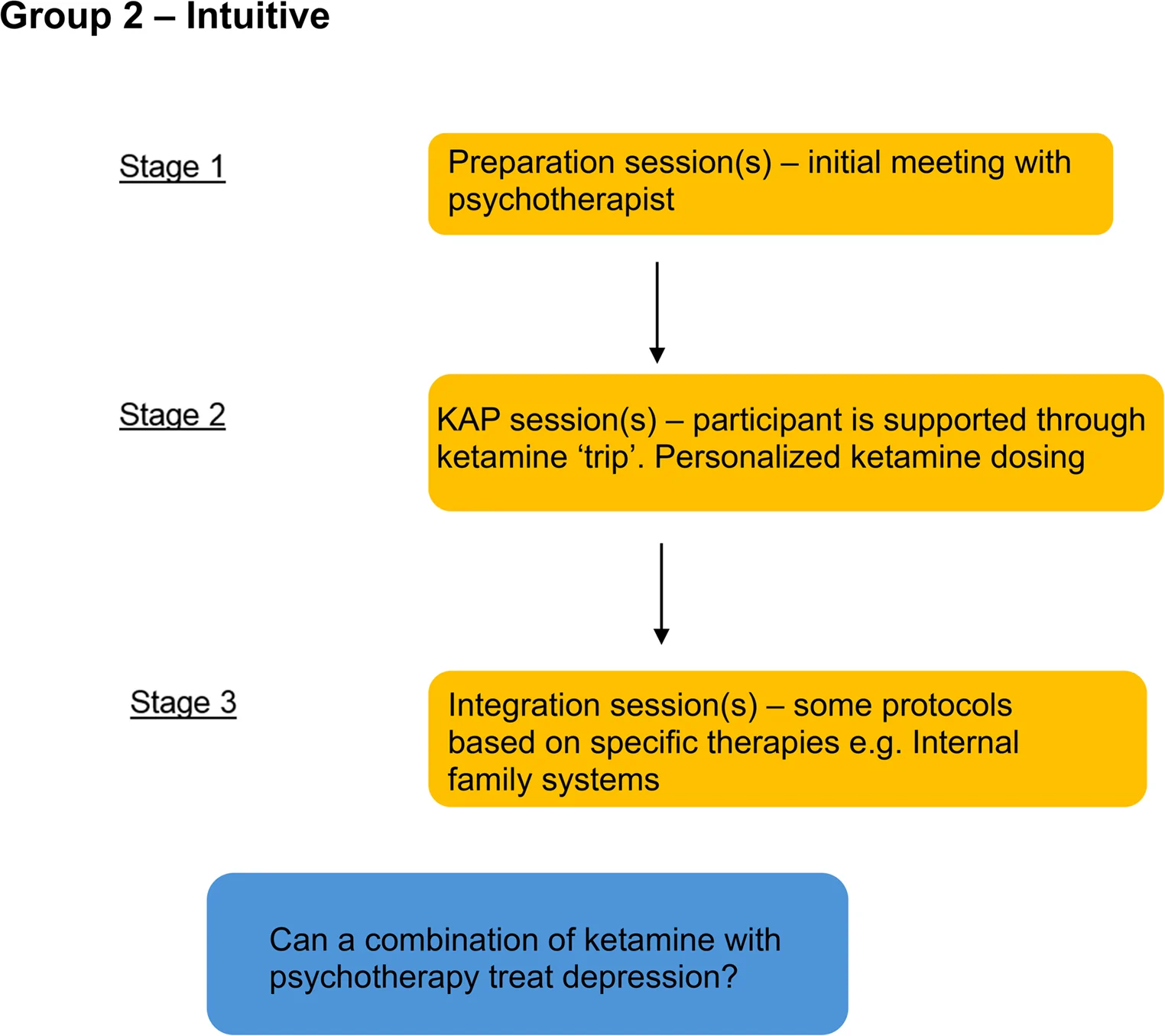
Flow diagram highlighting the different stages adopted by studies in group 2. Treatment consisted of preparation, KAP and integration sessions. This group focusses on, and uses, the subjective experience of ketamine and psychotherapy to lead treatment. Dark Yellow - Treatment Type; Blue – Study Aim. Abbreviations: Ketamine-Assisted Psychotherapy (KAP)

**Fig. 4:**
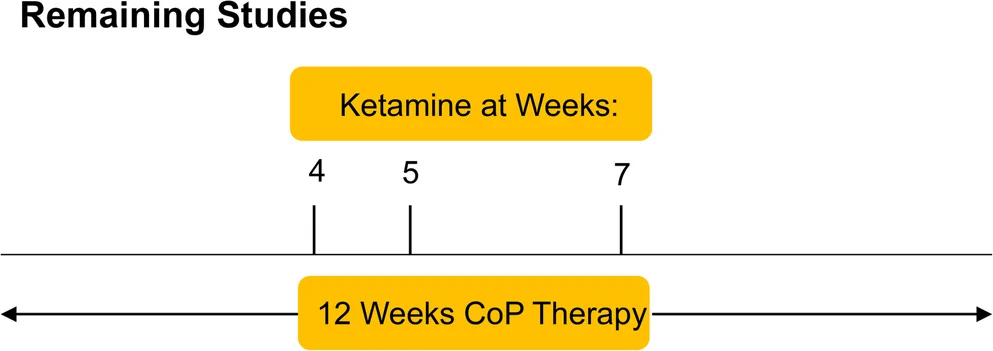
Diagram highlighting the treatment schedule adopted by the remaining studies (Dames et al. 2022; Tsang et al. 2023) Those who opted for additional ketamine received it at weeks 4, 5 and 7 of the 12-week CoP programme. Dark yellow: Treatment type. Abbreviations: Community of Practice (CoP)

Taking a bird’s eye view, the studies appear to loosely fall into two categories - one group subscribes to a prescribed model, whereas the other focuses on the intuitive experience of the individual. The first group has three studies (Wilkinson et al. 2017, 2021; Phillips et al. 2023) and group two has six studies (Dore et al. 2019; Montjoy 2022; Robinson et al. 2022; Oswin 2023; Willen 2024; Yermus et al. 2024). The remaining two studies (Dames et al. 2022; Tsang et al. 2023) do not fit perfectly into either but lean more towards the intuitive group. Figures 2, 3 and 4 outline an overview of the treatment stages for each group. Following this, a detailed table (see Table 2) highlighting the study characteristics and main findings of each paper is displayed. Finally, a narrative synthesis provides an in-depth description of important factors so one can go on to analyse whether KAP is an effective treatment for TRD.

**Table 2:** Studies included in systematic review, main characteristics, and key findings

Author and Year	Study Characteristics	Main Findings
Dames et al. 2022	• Community of Practice (CoP) therapy with adjunct ketamine at weeks 4, 5 and 7 (total 12-week programme) • *N* = 94 (total), TR-D *n* = 62 • Comorbidity present: Generalised anxiety disorder (GAD, *n* = 55); post-traumatic stress disorder (PTSD, *n* = 37)	In this **cohort-based case report**, when comparing baseline depression scores with those 1–2 weeks after completing the programme, **79%** of those with TR-D saw their **depression score** (as measured by **PHQ-9**) **fall into a milder category** or had a significant clinical improvement (> 5 point reduction).
Dore et al. 2019	• KAP sessions – supported ketamine trip; no mention of specific psychotherapy • *N* = 235 (total), TR-D *n* = 148 • Comorbidity present: Complex-PTSD (c-PTSD, *n* = 80; Attention deficit hypersensitivity disorder (ADHD, *n* = 65), PTSD (*n* = 65); GAD (*n* = 59), other anxiety disorder (*n* = 45), other mood disorder (*n* = 40), substance use disorder (SUD, *n* = 39), obsessive compulsive disorder (OCD, *n* = 15) • MA = 42.7; 48.9% female	In this **retrospective data analysis** (RDA), three KAP programmes from the United States (US) were evaluated. It was found that KAP significantly decreased depression scores (as measured by **Beck’s depression inventory (BDI)** (***p* < 0.0001**)). Further, the number of KAP sessions positively correlated with improvements in depression scores (**BDI: *p* < 0.01**; **Levine Rapid Depression Scale *p* < 0.001**), indicating that a greater number of sessions produces better results.
Montjoy 2022	• KAP sessions – supported ketamine trip with a mixture of Jungian, internal family systems and attachment theory delivered before, during and after ketamine treatment • TR-D *n* = 33 • Comorbidity present: PTSD (*n* = 21) • MA = 43 ($\:\pm\:$15); 63.6% female; 87.8% white	In this **RDA**, data from an outpatient clinic in the US where participants received **3 or 6 KAP sessions** in the last 12 months. A **significant decrease** in depression score (as measured by **PHQ-9**) was observed (***p*** **< 0.001**, d = 0.97) from pre- to post-KAP sessions. There was however no statistically significant interaction between change in depression severity and the number of KAP sessions (***p* = 0.714**) **– number of sessions does not impact change in depression score.**
Oswin 2023	• Ketamine only vs. KAP sessions – supported ketamine trip with CBT, ACT, dialectal behavioural therapy or EFT delivered during or 1 h after ketamine • TR-D *n* = 73; KAP *n* = 24; Ketamine only *n* = 49 • Comorbidity present: GAD, ADHD, PTSD, AUD (no specific values, but 10 participants had TR-D only) • MA = 40.5; 60.3% female	In this **RDA**, the difference in **PHQ-9** from baseline to post- treatment of participants who received KAP vs. those received ketamine only - **no significant difference** between the two groups (***p* = 0.068**) was found. When analysing the effectiveness of each treatment alone, both were found to significantly reduce PHQ-9 (KAP group: *p* < 0.001, d = 1.62); ketamine only group: *p* < 0.001, d = 1.85)
Phillips et al. 2023	• Multiphase: *Phase 1*: Ketamine double-blind crossover with midazolam; *Phase 2*: Ketamine only, responders move onto *Phase 3*: Ketamine only; *Phase 4*: BA only • TR-D *n* = 13 • Comorbidity present (panic disorder, *n* = 1; GAD, *n* = 3; agoraphobia, *n* = 2; social phobia, *n* = 1; OCD, *n* = 1) • MA = 46.9; 69.2% female	In this multi-phase **case series**, 10/13 participants completed the full course of BA (phase 4). There was a **significant improvement on BDI-II** (***p*** **= 0.007**) from pre- to post-BA. The 6 participants who began BA (phase 4) before relapsing sustained the AD effects of ketamine infusion; the remaining 4 participants had a more varied response to BA. Participants received an average of **14.8** ($\:\pm\:$1.3) BA sessions, with an average of **31.2** days ($\:\pm\:$24.8) between final ketamine infusion and BA initiation.
Robinson et al. 2022	• KAP sessions – combined ketamine and group therapy • *N* = 5 • Diagnosis = eating disorder with comorbid depression and anxiety disorders • Depression severity: 4 severe, 1 moderate (as per PHQ-9) • MA = 32.2, 100% female	In this **case series** participants received 4x weekly group KAP sessions. 4/5 participants experienced **clinically significant improvements on PHQ-9** (> 5 point reduction) **(mean change = −11.8, max = −23; min = −3) from baseline to 24-hour post final ketamine dose.
Tsang et al. 2023	• CoP therapy with adjunct ketamine at weeks 4, 5 and 7 • *N* = 57 (total) • MDD *n* = 43 • Comorbidity present: GAD (*n* = 34); PTSD (*n* = 27) • MA = 49.4 (SD = 13.8); 73.7% female; 84.2% white • Control: no ketamine (-KaT),	This **RDA** is a follow on from study **no.1**. There was a statistically significant reduction of mean change in **PHQ-9** scores from baseline to post treatment in both groups: • +KaT: mean change = −7.08 (95% CI: −9.25, −4.91, *p* < 0.001, d = 1.07) • -KaT: mean change = −6.21 (95% CI: −9.15, −3.27, *p* < 0.001, d = 1.02) There was however **no statistically** different change in PHQ-9 score when **comparing** + KaT and -KaT: • Adjusted for comorbidity, age, gender, and length of follow-up −0.78 (CI: −3.28, 1.72, *p* = 0.534)
Wilkinson et al. 2017	• Multiphase: *Phase 1*: Ketamine and CBT; *Phase 2*: CBT only • TR-D *n* = 16 • Mean age (MA) = 42.7 ($\:\pm\:$13.7); 75% female; 93.8% Caucasian	In **this open-label trial**, 50% of participants achieved a response ($\:\ge\:50\%$ MADRS) after the first ketamine phase. Following the **CBT only** phase, participants relapsed after a median value of 12 weeks – evidence that **CBT could sustain** the positive AD effects of ketamine infusions. A similar pattern was observed in both **MADRS and QIDS-SR-16**.
Wilkinson et al. 2021	• Multiphase: *Phase 1*: Ketamine only; *Phase 2*: Treatment as usual (TAU) or CBT • TR-D *n* = 42; 28 went onto phase 2 (ketamine responders, $\:\ge\:50\%$ MADRS) • MA = 46.6; 53.6% female; 92.9% Caucasian • Control = TAU group	In this **randomised clinical trial** (RCT), those who responded to ketamine received an additional 14 weeks of CBT or TAU. Despite a moderate effect size (d = 0.65), time-by-treatment interaction was **not significant for MADRS** (***p*** **> 0.05**) but was **significant** when measured by **QIDS-SR-16** (F = 4.58; *p* = 0.033, d = 0.71).
Willen 2024	• KAP sessions – supported ketamine trip; no mention of specific psychotherapy • *N* = 38; 19 had active MDD diagnosis • Comorbidity present: no exact figures, but mentions PTSD in discussion • No sample characteristics	In this **RDA**, data was analysed from an outpatient clinic in the US providing **KAP**. Depression severity, as per **PHQ-9**, of the total sample significantly decreased after treatment (***p*** **= 0.00, d = 0.68)**. Those with severe depression (baseline PHQ-9 score 20–27) benefitted most substantially, with a significant reduction in PHQ-9 from pre- to post- treatment and large effect size (***p*** **= 0.01**, d = 2.49). An average of **6 KAP** sessions were received.
Yermus et al. 2024	• Ketamine only sessions followed by psychotherapy integration sessions (protocol based on motivational interviewing, BA and trauma-informed therapy) • *N* = 1806 started; 346 at 3 months; 94 at 6 months • MDD primary diagnosis *n* = 177 • Comorbidity present: Anxiety (*n* = 157), bipolar disorder (*n* = 5), eating disorder (*n* = 12), OCD (*n* = 17), PTSD (*n* = 156), SUD (*n* = 27), other (*n* = 48) (values represent those who had follow up data *n* = 616) • MA = 42 (SD = 12); 52% female; 78.4% white	In this **RDA**, the lasting effects of KAP were analysed at **1-**,** 3-**,** and 6-months** post-treatment. Average number of **ketamine doses** was **4** ($\:\pm\:$3) and mean number of **integration sessions** was **3** ($\:\pm\:$2). There was a significant reduction on **PHQ-9** at 1 month (d = 0.5), 3 months (d = 0.85) and 6 months (d = 0.73) (all *p* < 0.0001)

As per the search strategy (see Fig. 1) a total of eleven studies were identified, with a cumulative total of 2412 participants. The type of study included in this analysis varies and includes: one cohort-based case report (Dames et al. 2022), one open-label trial (Wilkinson et al. 2017), one randomised clinical trial (Wilkinson et al. 2021), two case series (Robinson et al. 2022; Phillips et al. 2023) and six retrospective data analyses (Dore et al. 2019; Montjoy 2022; Oswin 2023; Tsang et al. 2023; Willen 2024; Yermus et al. 2024), with three of these being PhD theses (Montjoy 2022; Oswin 2023; Willen 2024). Due to the nature of study types present in this review – there was no attempt to blind participants in most papers - it was decided bias could not be measured using an online risk-of-bias tool (Sterne et al. 2019).

The reporting standards of several studies (Wilkinson et al. 2017, 2021; Dames et al. 2022; Robinson et al. 2022; Phillips et al. 2023) were measured using the CONSORT checklist and can be found in the supplementary material. The CONSORT checklist (2010) consists of 25 items that looks to evaluate the standard of reporting of randomised controlled trials (Schulz et al. 2010). However, the items in the checklist pertain to most types of trial (Schulz et al. 2010). Therefore, it will be applied the RCT conducted by Wilkinson and Colleagues (2021), with questions regarding randomisation omitted for the remaining studies (Wilkinson et al. 2017; Dames et al. 2022; Robinson et al. 2022; Phillips et al. 2023).

The remaining studies (Dore et al. 2019; Montjoy 2022; Oswin 2023; Tsang et al. 2023; Willen 2024; Yermus et al. 2024) are categorised as retrospective data analyses (RDA) and therefore cannot be measured by the CONSORT checklist. To the authors knowledge, there is not a validated tool for measuring the quality of reporting of this study type.

### Summary of main findings

Bringing the main findings together, largely promising results were found. Due to the heterogeneity of the studies involved, there are different aspects of the findings to consider. Studies that solely examined the effectiveness of KAP in reducing depressive symptoms (Dore et al. 2019; Dames et al. 2022; Montjoy 2022; Robinson et al. 2022; Willen 2024) reported significant reductions. Studies in the prescribed group examined the potential of psychotherapy – CBT and BA - in sustaining the AD effects of ketamine (Wilkinson et al. 2017, 2021; Phillips et al. 2023). These all reported that psychotherapy following ketamine treatment can prolong the AD effects of ketamine. One study (Yermus et al. 2024) examined the longevity of positive effects of KAP at regular time intervals up to 6 months, reporting sustained benefits at 1-, 3-, and 6-months post final KAP session.

**Fig. 5:**
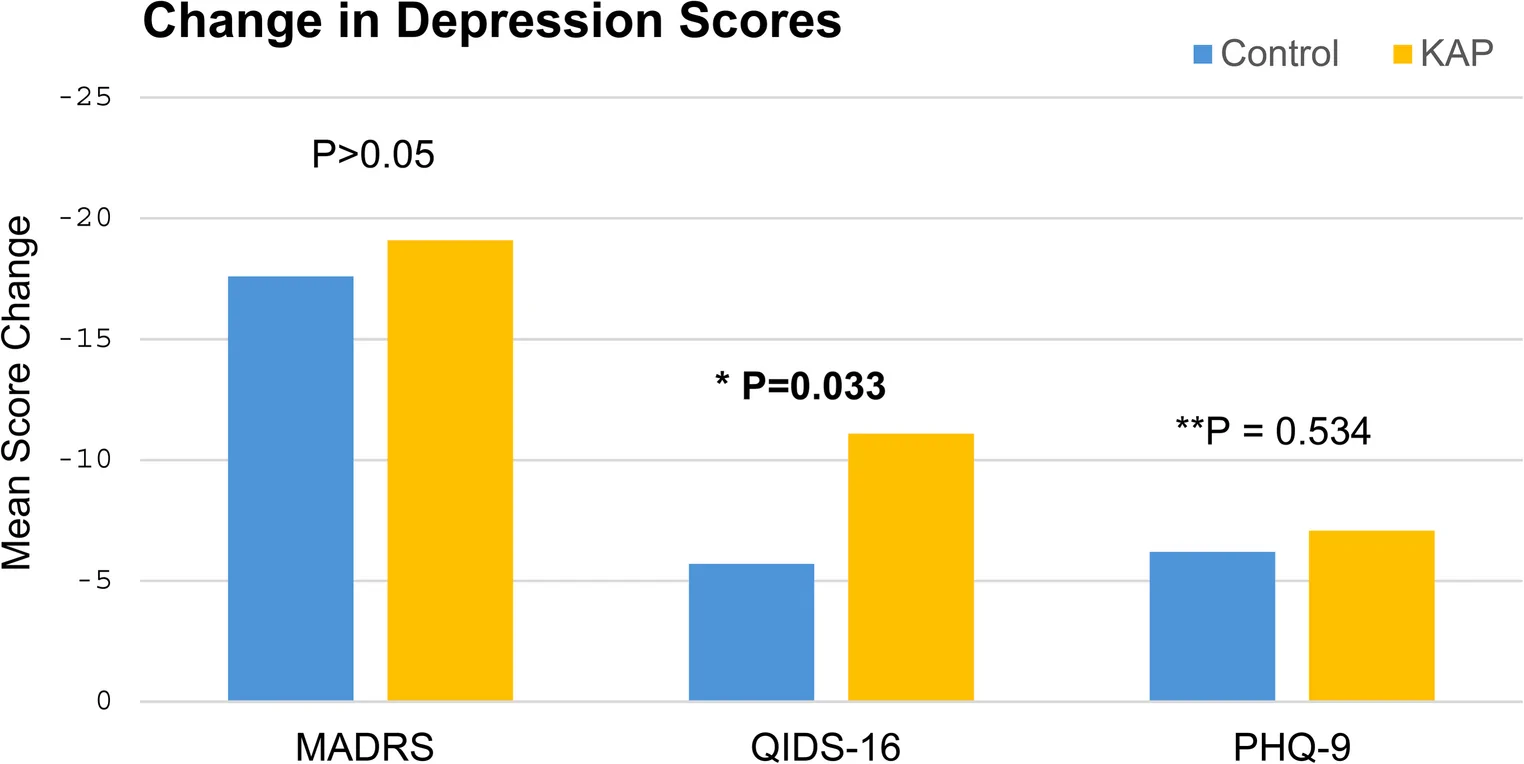
Bar graph representing the average change in depression score from pre-to post-treatment as measured by MADRS and QIDS-16 (Wilkinson et al. 2021) and PHQ-9 (Tsang et al. 2023). * Indicates a significant reduction in depression score. ** reporting adjusted p-value

Those studies which had a control group comparing ketamine only against ketamine with psychotherapy (Oswin 2023; Tsang et al. 2023), found that there was no significant difference between the treatment options (despite individual group analysis producing significant results). One study used a control group in the second phase, comparing the effectiveness of additional CBT against treatment as usual (TAU) – significant results were observed when using a self-rating (SR) scale, but not when using a clinician administered (CA) questionnaire (Wilkinson et al. 2021). The average change in depression score for the control and active groups from the studies conducted by Tsang and Colleagues (2023), and Wilkinson and Colleagues (2021) are represented in Fig. 5. The author(s) calculated the average score changes for one study included in Fig. 5 (see supplementary material – Figure S2). There was no data available on the mean change of participant scores in the remaining study (Oswin 2023) so this was not included in Fig. 5.

Three studies reported some valuable additional analysis: the number of KAP sessions had **no** impact on outcome (Montjoy 2022), the number of KAP sessions ***did*** have an impact on outcome (Dore et al. 2019), and those with the most severe form of MDD had the most significant benefit from KAP (Willen 2024).

Overall, KAP is effective in sustaining the AD effects of ketamine infusions and can successfully reduce the symptoms of TRD. It appears to work best in those with the most severe and chronic forms, yet the effectiveness of KAP against a control group is not necessarily supported.

### Correlation between type of intervention and success rates

It is challenging to visualise any correlation between treatment type and success in reducing depressive symptoms. This is principally due to the variety of treatment types and schedules; there is almost no similarity between studies for route and dose of ketamine administration, or the type and timing of psychotherapy. All studies report positive findings, implying both treatment types (ketamine followed by psychotherapy and combining within the same session) are beneficial.

### Ketamine administration and dosing

**Fig. 6:**
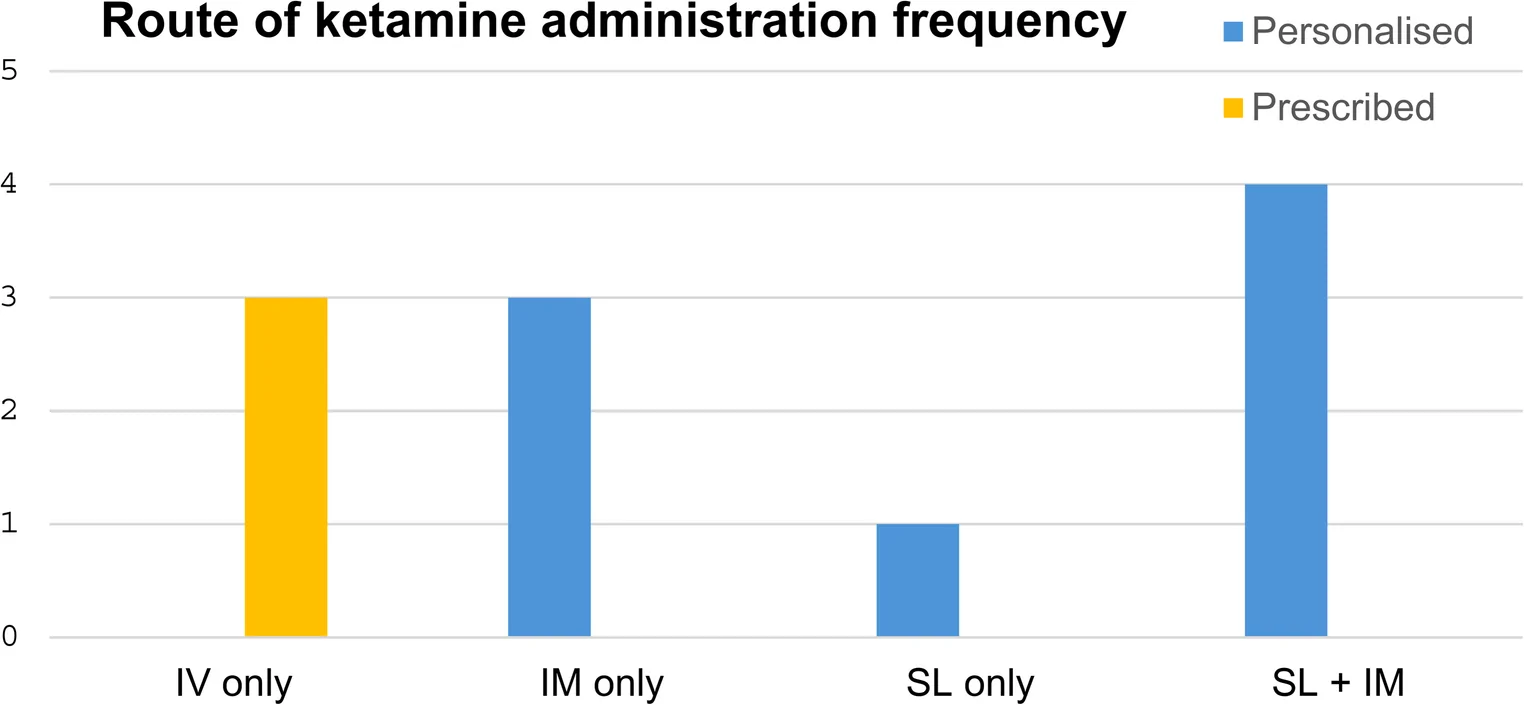
Bar chart showing the number of times each route of ketamine administration was used. The bars are colour-coded to differentiate between dosing ideology. A personalised dosing schedule was adopted in the intuitive group

The route of administering, and dose of, ketamine given to participants varied greatly between studies. Four different routes were observed: intravenous (IV), intramuscular (IM), sublingual troche (SL), or a combination of SL and IM (see Fig. 6). Those in the prescribed group (Wilkinson et al. 2017, 2021; Phillips et al. 2023) administered a dose of 0.5 mg/kg over 40 min to participants via IV. Despite this similarity, the schedule in which participants received the drug was different. Both studies conducted by Wilkinson and Colleagues (2017; 2021) gave subjects two doses per week for two or three weeks. However, the remaining study in the prescribed group employed a multi-phase approach: phase 1 included one infusion administered in a randomised double-blind crossover with midazolam, phase 2 was six infusions in two weeks, and phase 3 consisted of four weekly infusions (Phillips et al. 2023).

One study administered ketamine via SL weekly or biweekly for 3–16 sessions (min = 3; Max = 16, Average = 6), with no mention of dose (Willen 2024). The RDA conducted by Oswin (2023) references the protocol outlined by Dore and Colleagues (2019) (see Table 4) but fails to provide dose ranges. One study gave two ketamine injections per session (Montjoy 2022). The studies in the intuitive group used IM injections, SL, or a combination of SL and IM are summarised in Tables 3 and 4. Interestingly, these all implemented a personalised dosing schedule based on response to, and experience of, the first dose (see Fig. 6).

**Table 3:** **IM** ketamine dose and schedule

Study	Dose	Schedule
Dames et al. 2022	1–1.5.5 mg/kg	One dose given at weeks 4, 5 and 7
Montjoy 2022	0.3 mg/kg with titration up to 1 mg/kg **OR** Session total: 20–70 mg	2x injections per session, second given 12 min after first. 3–6 sessions over 1–3 weeks (36.36% 3 sessions; 60.6% 6 sessions; 0.03% 5 sessions)
Robinson et al. 2022	25–100 mg	One dose given per session (4 sessions in total)

**Table 4:** **SL** and **IM** ketamine dose and schedule. Percentages represent the proportion of the total sample receiving ketamine via that route

Study	Dose	Schedule
Dore et al. 2019 **(** Oswin 2023**)**	SL: 200–250 mg (38.5%) **and/or**: IM: 80–90 mg (61.5%) Applies to study no. 2 sample only	Varying frequency (depending on diagnosis) between 1–25 sessions, spread over variable time periods (6 over 2–3 weeks)
Tsang et al. 2023	SL: 100–300 mg in first session, **then**: IM: 1–1.5.5 mg/kg	One dose given at weeks 4, 5 and 7
Yermus et al. 2024	SL: 200–500 mg (32%) **or**: IM: 25–100 mg (68%)	4–6 doses (mean = 4, 3) (12% received > 6; 24% received 1)

### Type of psychotherapy

The type of psychotherapy given to participants also varied. Those in group 1 used therapies with a well-documented evidence base like CBT (Wilkinson et al. 2017, 2021), or BA (Phillips et al. 2023). Two studies (Dames et al. 2022; Tsang et al. 2023) used a group community of practice (CoP) therapy. CoP aims to help individuals who share a similar ideology or concern learn how to deal with this more effectively; to help build resilience (Wegner-Treyner & Wegner-Treyner, 2015). This was carried out in an online, group format in both studies (Dames et al. 2022; Tsang et al. 2023). The remaining six studies in group 2 (Dore et al. 2019; Montjoy 2022; Robinson et al. 2022; Oswin 2023; Willen 2024; Yermus et al. 2024) describe a therapeutic methodology that does not subscribe to traditional talking therapies. Initially, the therapist will have a preparatory meeting with the participant to build rapport and trust and inform them of what to expect from the ‘KAP session(s)’. During KAP sessions, participants take the drug wearing an eye shade and have access to music, and the therapist is present for any necessary reassurance and support. These sessions can last for up to 4 h and can be done individually (Dore et al. 2019; Montjoy 2022; Oswin 2023; Willen 2024; Yermus et al. 2024) or in group settings (Robinson et al. 2022). Following this, integration session(s) are offered. A combination of therapies was used in these integration sessions. One study used Jungian, internal family systems and attachment theory (Montjoy 2022); another used BA in conjunction with motivational interviewing and trauma-informed practice (Yermus et al. 2024). Those who elected to receive ketamine, in addition to CoP therapy (Dames et al. 2022; Tsang et al. 2023) also had therapeutic integration sessions (within 36 h of receiving the drug).

### Delivery of ketamine and psychotherapy – before, after or concurrent?

One aspect in which groups 1 and 2 differ is the timing of ketamine and psychotherapy. As mentioned above, several papers (Dore et al. 2019; Montjoy 2022; Robinson et al. 2022; Oswin 2023; Willen 2024; Yermus et al. 2024) describe KAP sessions with preparatory and integration sessions. There was little information regarding the time between KAP and integration sessions. One study from this cluster states that psychotherapy was delivered during preparation, intra-KAP and post-KAP (Montjoy 2022). There is more information from studies in group 1, where the two treatments were delivered in separate sessions. In the studies using CoP therapy, participants received this within the first 36 h following the ketamine dose (Dames et a., 2022; Tsang et al. 2023). Phillips et al. (2023) hoped to deliver BA to participants within 10 days following ketamine. However, results showed that the average time was 31.2 ($\:\pm\:$24.8) days between treatments, with 7/13 participants starting within 16 days. Despite this, there was no apparent correlation between the time interval of ketamine, BA and baseline depression scores (*r*= −0.13, *p*= 0.966). Finally, Wilkinson et al. (2017) employed a two-phase approach, the first consisting of ketamine and CBT concurrently but separately (24–48 h after infusion), followed by a CBT only phase. For this study (Wilkinson et al. 2017) and the RCT (Wilkinson et al. 2021), there is no information regarding the time between final ketamine infusion and initiation of psychotherapy.

### Number of sessions given to participants

The number of treatment sessions given to participants is different for each group. Generally, those in the prescribed group (Wilkinson et al. 2017, 2021; Phillips et al. 2023) experience a larger number of sessions, albeit spread across two phases. Phillips and Colleagues (2023) deliver 11 ketamine infusions (over an average of 8.6 ($\:\pm\:$2) weeks) followed by an average of 14.8 ($\:\pm\:$1.3) BA sessions. The two studies conducted by Wilkinson et al. (2017; 2021) have similar scheduling and number of sessions. Four ketamine infusions (followed by separate CBT session, see Fig. 2) in the first stage then 12 CBT sessions in the second (Wilkinson et al. 2017); six ketamine infusions over 3 weeks followed by 14 CBT sessions (Wilkinson et al. 2021).

For the intuitive group (Dore et al. 2019; Montjoy 2022; Robinson et al. 2022; Oswin 2023; Willen 2024; Yermus et al. 2024) there are 3 types of sessions on offer to participants: preparatory, KAP and integration (outlined in Fig. 3). Apart from one study where session number ranged between 1 and 25 (Dore et al. 2019), studies in this group offered a much smaller number of KAP sessions – between 3 and 6 (see Tables 3 and 4). The remaining studies (Dames et al. 2022; Tsang et al. 2023) offered a 12-week programme, with 3 adjunct ketamine sessions for those who selected it. There was very little information regarding the number of preparatory and integration sessions – most provide no data (Dore et al. 2019; Montjoy 2022; Robinson et al. 2022). In the RDA performed by Willen (2024), the centre offering treatment recommended 1–3 preparation sessions followed by an initial 3 KAP sessions (additional 3 offered if necessary) with an integration session offered within 48 h of each KAP. There is no data regarding the number of preparatory sessions from the RDA performed by Yermus and Colleagues (2024), but the authors report that integration sessions were offered after doses 1 and 2 then after each 2 subsequent doses (mean = 3 ($\:\pm\:$2).

### Depression questionnaires

Several depression questionnaires were used throughout the studies. The most common scale used was the SR Patient Health Questionnaire (PHQ-9), which appeared in eight studies (Dore et al. 2019; Dames et al. 2022; Montjoy 2022; Robinson et al. 2022; Oswin 2023; Tsang et al. 2023; Willen 2024; Yermus et al. 2024). Two studies (Wilkinson et al. 2017, 2021) used a combination of CA and SR scales - Montgomery–Åsberg Depression Rating Scale (MADRS) and Quick Inventory of Depressive Symptomology 16 (QIDS-16). The SR Beck’s Depression Inventory (BDI) appeared in two studies (Dore et al. 2019; Phillips et al. 2023); the Levine Depression scale was used as an additional follow-up measure (Dore et al. 2019).

### Presence of comorbidity

Two studies used a population where no comorbidity was present, i.e. participants had TRD only (Wilkinson et al. 2017, 2021). In the remaining nine, diagnoses including post-traumatic stress disorder (PTSD), eating disorders (ED) and obsessive-compulsive disorder (OCD) are seen in varying numbers. The most frequent comorbid diagnosis was anxiety disorder (GAD), appearing in six studies, closely followed by PTSD which appears in five.

## Discussion

This review aimed to analyse the literature that exists regarding KAP as a treatment for TRD. Largely positive results were reported. However, when KAP was compared to TAU, ketamine only or psychotherapy only, results showed there was no significant difference between groups, implying that KAP is no more or less effective than these options. No consistent KAP protocol emerged, making correlation between treatment aspect and therapeutic outcome challenging.

### Difference in treatment ideology

Before contextualising the results of this review, it is worth understanding in more detail the difference in treatment ideology between groups 1 and 2. The prescribed group operates under what has been referred to in the literature as a “plasticity-oriented” rationale (Mathai et al. 2022). Ketamine is used as a “psychoplastogen” – a molecule producing rapid and measurable changes in plasticity (Olson 2018) – to improve outcomes in additional psychotherapy. Talking therapies in this group are delivered in a separate stage to ketamine, and there is relatively less emphasis on, or encouragement of, drug-induced experiences (Mathai et al. 2022). This contrasts greatly with the rationale of the remaining studies. Ketamine is treated as a psychedelic drug with dissociative properties, and the subjective experience of the participant is critical in therapeutic outcomes. In the literature this has been referred to as an “experience-oriented” treatment modality (Mathai et al. 2022), which is challenging to specifically define and create a protocol for (Wolfson and Vaid 2024).

### Prescribed group: Ketamine protocol

The prescribed group (Wilkinson et al. 2017, 2021; Phillips et al. 2023) use a standard ketamine protocol of 0.5 mg/kg over 40 min (delivered via IV), with sessions generally biweekly for two or three weeks. This has been a common route and dosage since two early control trials (Berman et al. 2000; Zarate et al. 2006; Andrade 2017). However, the now established dose range for IV ketamine in treating MDD is 0.5-1.0.0.0 mg/kg over 40–60 min twice weekly for 2 weeks (McIntyre et al. 2021). Despite using the lower end of established dosing, the prescribed group seems to follow an evidence-based ketamine protocol.

### Prescribed group: Psychotherapy protocol

On completion of the first ketamine stage, participants in this group went on to receive BA (Phillips et al. 2023) or CBT (Wilkinson et al. 2017, 2021). Both are recommended to treat MDD as they have an established evidence base (NICE, 2022). The number of CBT sessions delivered was 12 (Wilkinson et al. 2017) and 14 (Wilkinson et al. 2021), with an average of 14.8 ($\:\pm\:$1.3) BA sessions given (Phillips et al. 2023). Again, these are close to the suggested range as per the NICE guidelines – 16 sessions for individual CBT and between 12 and 16 for BA (NICE, 2022). Despite significant improvements in depression scores reported by these studies, increasing both the dose of ketamine (up to 1.0 mg/kg), and the number of psychotherapy sessions, could be a way to improve success rates even further.

### Prescribed group: Neuroplasticity

At the core of the prescribed group ideology is the idea that ketamine induces a state of neuroplasticity which can be harnessed to improve outcomes in psychotherapy. Therefore, being aware of the length of time ketamine increases neuroplasticity is central in the success of such treatments. One study aimed to define this period, finding that there was elevated plasticity potential 4 h after taking the drug, but this had dissipated by 12 h post infusion (Wu et al. 2021). However, contrary to this point, a different study states that the plasticity induced by ketamine can last for up to 24 h post infusion, with potential for this to continue well beyond that time point (Kopelman et al. 2023). Relating this to the studies in this review, where an average of 31.2 ($\:\pm\:$24.8) (Phillips et al. 2023) days was taken between ketamine and BA, it is unlikely the neuroplastic state induced by ketamine would be still active. There was no information regarding the time between ketamine and CBT treatments (Wilkinson et al. 2017, 2021). Ultimately, it appears that having the ketamine and talking therapy stages as close together as possible would harness the neuroplastic state induced by ketamine. There is a need for more research quantifying the length of time that ketamine induces neuroplasticity in the brain, as this will allow for more accurate timing with talking therapy, improving outcomes.

### Intuitive group: Ketamine protocol

The studies in the intuitive group (Dore et al. 2019; Montjoy 2022; Robinson et al. 2022; Oswin 2023; Willen 2024; Yermus et al. 2024) use the aforementioned “experience-oriented” rationale for KAP. Of these, one seminal paper published by Dore and Colleagues (2019) sets the stage for KAP to be viewed in this way. As subjective experience is fundamental in the success of group 2, it follows that these studies have a personalised ketamine dosing protocol. Participants start at a low dose and can escalate this depending on their tolerance and desired effect of the drug (Dore et al. 2019). Three factors dictate the experience of ketamine: the route of administration, dose, and the individual’s sensitivity to the drug (Wolfson and Vaid 2024). It appears that using a mg/kg dosing approach is not linearly associated with the effect on the recipient (Wolfson and Vaid 2024), reiterating the need to start at a low dose and titrate up. Perhaps because of this desire for a person-centred and “experience-oriented” approach, the ketamine protocols for group 2 are incredibly diverse. A variety of routes of administration and dosing ranges can be seen, with almost no similarities between papers.

IV was not used in this group, with IM and SL or a combination, being utilised. IV ketamine has an almost complete bioavailability (McIntyre et al. 2021), in that nearly 100% of ketamine administered in this way will be absorbed into the bloodstream and influence the individual. The bioavailability of IM is thought to be close to that of IV, but SL has a much lower rate of 20–30% (McIntyre et al. 2021), implying that less ketamine will be absorbed and therefore have an influence. The studies using SL in group 2 report positive findings, despite having a lesser influence of ketamine. This highlights the difference in tolerance that subjects may have to ketamine, and the positive impact that taking ketamine within a therapeutic environment can have on outcomes.

### Intuitive group: Protocol

KAP as an “experience-oriented” modality, as seen in the intuitive group, was first observed in 1992 when Krupitsky and Colleagues used ketamine-enhanced psychotherapy (KEP) to treat alcoholism (Krupitsky et al. 1992). Here, there was a focus on creating a safe and comfortable environment for participants. Indeed, it is theorised that the therapeutic effects of mind-altering drugs cannot be explained by their pharmacological properties alone (Pahnke et al. 1970). This could explain the difference in route of administering ketamine between groups. The intuitive group focuses on the importance of “set” and “setting” and uses less invasive routes (SL or IM), whereas the prescribed group uses IV which needs to be delivered in a more clinical setting. Despite being treated as such, it is worth noting that ketamine is not a classic psychedelic. Ketamine is an anaesthetic sedative with opioid and glutamate action (Williams et al. 2018), whereas classic psychedelics require 5HT2A activation (Gonzales-Maeso et al. 2007; Ly et al. 2018).

The structure of preparation, dosing and integration sessions was also first noted with ketamine in 1992 when Krupitsky and Colleagues used KEP to treat alcoholism (Krupitsky et al. 1992). The number of KAP sessions in the studies of group 2 largely fall between 3 and 6 with little information regarding the number, content and timing of preparation and integration sessions. One would assume these were an integral part of the overall KAP process, and so more information regarding these sessions is necessary in the future. The type of therapy given during sessions can be moulded to the disorder being treated. A direct therapeutic approach was used by Krupitsky and Colleagues (1992) that had a focus on sobriety, whereas in group 2 the aim was for participants to feel reassured & supported in the dosing sessions. The type of psychotherapy in the integration sessions varied – Jungian to motivational interviewing. This again perhaps ties in with the person-centred approach observed by the studies in group 2.

There are some general psychotherapy points that apply to both the prescribed and intuitive groups. The number of sessions in the prescribed group was much more than that of the intuitive group − 12–16 psychotherapy sessions in group 1 compared to 3–6 in group 2. However, if more information was available on the number of preparation sessions in group 2, a more accurate comparison could be made. In non-psychedelic assisted psychotherapy, the number of sessions is not associated with treatment effects in adult depression (Ciharova et al. 2024), but the number of sessions per week is (Cuijpers et al. 2013). The “alliance” between therapist and client – the collaborative and supportive relationship – has been shown to positively correlate with success (Fluckiger et al. 2018). This point has been echoed in the KAP protocols of the intuitive group, where an importance has been put on making participants feel safe. These points may not be directly applicable to the studies in this review as they pertain to psychotherapy in the absence of ketamine. However, such robust correlations could be used to inform greater practice in future KAP studies. If possible, having more sessions within a week, where building alliance and trust between the client and therapist has been of high importance, may be beneficial.

### Depression questionnaires

The main type of questionnaire used to evaluate changes in depressive symptoms was the SR PHQ-9. Only two studies administered a CA questionnaire, the MADRS (Wilkinson et al. 2017, 2021). The PHQ-9 is a well-established, reliable and accurate tool for measuring the presence or absence of depression, but not for quantifying the severity of symptoms (Kroenke et al. 2001; Lin et al. 2014). Other disadvantages of SR scales include the impact of positive response bias and social desirability effects (Moller 2014). These points are especially important to note as all but one of the studies are open label, where participants are aware of their treatment arm and expected effects/benefits. Interestingly, one study which used both a SR and CA (Wilkinson et al. 2021) reported conflicting results - significant improvement using the QIDS-SR-16 but not with the CA MADRS (see Fig. 5). Due to the nature of treatment arms, blinding was challenging in this study and could have affected SR scores.

### Confounding factors

As well as the type of questionnaire used, another explanation for the similarity in outcome observed, where additional CBT was not more successful than TAU in sustaining ketamine treatment, is the absence of childhood trauma within the sample population (Wilkinson et al. 2021). Psychotherapy is known to be more beneficial than AD monotherapy in those with TRD and a history of childhood trauma (Nemeroff et al. 2003). There was no comorbidity with PTSD within the sample population, and on the assumption that this equates to an absence of early life stress, could in part explain why additional CBT was not more successful than TAU. There was little information pertaining to history of early life stress (ELS) in the remaining studies, but the impact ELS has on treatment outcomes with psychotherapy could be a crucial element to consider in future studies.

### Control groups

Three studies compared the success of KAP against a control group. These were KAP against ketamine alone (Oswin 2023), CoP therapy against CoP with three additional ketamine sessions (Tsang et al. 2023), and CBT against TAU after ketamine therapy (Wilkinson et al. 2021). Despite individual analysis of each group yielding significant results, none of the studies found a significant difference between groups, implying that one was no more effective than the other. Both treatment arms were as successful as each other (Oswin 2023); the addition of ketamine to CoP therapy was no more successful than CoP alone (Tsang et al. 2023); and TAU after ketamine treatment was just as successful in sustaining its AD effects (Wilkinson et al. 2021) (as measured by MADRS). These contrasting results make it difficult to discern which aspects of KAP are effective but also does not support the conclusion that KAP is more effective than psychotherapy or ketamine treatment alone.

These results lead to one to question whether there is a need for the addition of psychotherapy to ketamine treatment at all. Ketamine as a solo treatment has a strong evidence base for alleviating depressive symptoms and improving suicidal thoughts (McGirr et al. 2015; Marcatoni et al. 2020; Nikolin et al. 2023; Shen et al. 2024). However, the need for maintenance doses (Daly et al. 2019; McMullen et al. 2021), and the largely unknown impact of this, calls for a way to improve or sustain ketamine treatment. The addition of psychotherapy was hoped to improve or sustain ketamine’s effects. However, the results of this review do not support this; it is not clear whether adding psychotherapy adds any benefit to ketamine treatment. Ketamine is already more expensive than AD treatment, limiting its accessibility (Ross and Soeteman 2020) and one can assume that the inclusion of psychotherapy will only add to this cost.

### Overall study quality

The overall quality of the studies included in this review is relatively low. There is a distinct lack of comparison groups and adequately powered sample sizes. It should be noted that the populations of studies are overwhelmingly female and white, making generalisability of results challenging. Following this, several of the studies are data analyses of KAP clinics (Montjoy 2022; Robinson et al. 2022; Oswin 2023; Willen 2024; Yermus et al. 2024) where the protocol was based on the seminal paper published in 2019 (Dore et al. 2019). To the authors knowledge, it is the first paper that describes results of KAP sessions where dissociative and psychedelic experiences are seen as crucial in treatment outcomes. This paper presents data from clinics where KAP was used to treat various psychiatric conditions. No concrete protocol was published, and there appears to be a lack of evidence base for the published methods. Subjective experience is critical in this paper (Dore et al. 2019) and has been used to inform practice, with a bidirectional flow of information between participant and therapist. It appears to be the first paper documenting such a treatment modality and ideology, and so allowances can be made if it was being treated as an initial study. However, it is concerning that this clinical evidence, and not trial data, is forming the basis of treatment in ketamine clinics throughout the Americas.

It is also worth emphasising that many of the studies group did not provide key information on the type of therapy delivered to participants. In the first group, simply noting that either CBT or BA was adopted, without a therapist manual, does not give the reader enough information to ensure standardisation of treatment. Following this, those studies in the second group lack information on: the number of preparatory and integration sessions, what was done within the different sessions, or how therapists were trained. Essentially, the reader is unaware of the full study protocol, whether there was any effort to standardise treatments, or what therapy was delivered to participants. This represents a major limitation of the studies and prevents proper interpretation and comparison of the results obtained.

### Blinding procedures

As discussed above, all but one of the studies in this review are open label, meaning participants were completely aware of their treatment and any anticipated effects. In all treatment using mind-altering drugs, it is challenging to fully blind participants due to the profound psychoactive properties of the drugs (Szigeti and Heifets 2024). This poses a unique challenge to researching these compounds. Even when active placebos have been used in an attempt to blind participants, it is likely (in over 90% of occasions) they will be able to correctly guess their treatment arm (Bogenschutz et al. 2022). Using ketamine poses a distinct challenge in blinding of participants, but the lack of blinding throughout the studies in this review could have influenced the overwhelmingly positive results.

### Expectancy bias

Expectancy refers to a set of cognitions about the likelihood of future events (e.g. response to treatment) (Petrie and Rief 2019). Expectancy bias therefore occurs when an individual’s expectation about a drug or treatment can influence outcomes (Williams et al. 2012). Part of KAP in the intuitive group is preparation sessions where the client will be educated about dosing and the potential impacts/effects of the drug – i.e. explaining what could happen. Expectancy has been found to moderate the strength of the placebo effect (Bjorkedal and Flaten 2011; Howe et al. 2017), implying that if participants are aware of how they “could” react, they are more likely to have a false positive result. Being cognisant of this, one group of researchers conducted a triple-blinded experiment in which ketamine or a placebo was given to participants under general anaesthesia (Lii et al. 2023). No greater effect of single dose ketamine was observed over the placebo (Lii et al. 2023), suggesting that expectancy could be responsible for a large portion of treatment effect. Expectancy is a dynamic phenomenon and indeed has the potential to change between dosing sessions; the experience of the first session could affect the next (Szigeti and Heifets 2024). Bringing all of this together, in psychedelic research it is challenging to appropriately blind participants and eradicate expectancy bias. When applying this to the studies in this review, where there was little attempt to minimise such phenomena, it is almost impossible to discern how much impact the treatment truly had on individuals.

### Set and setting

One area that should also be considered is the impact of “set and setting” (S&S). It represents a further difference between the two groups – the intuitive group reference the importance of making participants feel safe and comfortable through adaptation of S&S, whereas the prescribed group adapt a more clinical approach with little to no reference to S&S. It is known that extra-pharmaceutical (EP) contexts – such as the environment, music, attitudes and social interactions – have critical influence on psychedelic drugs (Hartogsohn 2017; Dore et al. 2019; Pronovost-Morgan et al. 2025). One would assume the difference in attitude towards S&S of each group would impact the results. However, the prescribed and intuitive group both report largely positive findings. Therefore, discerning the true impact that S&S had on results is challenging, and is indeed compounded by a lack of reporting on such EP factors. This problem is not unique to the studies of this review; it is universal to the psychedelic research community. The recent publication of a set of guidelines – the Reporting of Setting in Psychedelic Clinical Trials (ReSPCT) – is composed of 30 items that reports on different aspects of the setting in which participants take the drug (Pronovost-Morgan et al. 2025). It is hoped these new guidelines will encourage transparency and validity when examining the effectiveness psychedelics.

### Future directions

It is apparent from this review that there is a distinct lack of high-quality studies examining the relationship between ketamine, psychotherapy and therapeutic outcomes in TRD. There is a need for randomised trials with large, diverse, sample sizes where attempts are made to minimise expectancy bias. Further, the newly defined ReSPCT guidelines offer a framework in which the true impact of external factors, such as setting, may have on outcomes.

More research is necessary to quantify the timeframe in which ketamine induces neuroplasticity. Knowledge of this will permit full capitalisation of such a state and hopefully produce more sustained, successful outcomes. It also seems that treating KAP in a similar way to other PAP yields positive results, but current understanding is based on a personalised and subjective rationale, making expansion of such treatment challenging. Each component of KAP – the ketamine route, dose and frequency, along with which type of psychotherapy – needs to be trialled with control groups to establish the most effective combination for TRD. It is hoped that following such future work, a KAP protocol could be created. Ketamine as a solo treatment has an extensive evidence base for treating depression (McGirr et al. 2015; Marcatoni et al. 2020; Nikolin et al. 2023; Shen et al. 2024), but the intricacies of combining it with psychotherapy, both in an “experience-oriented” and “neuroplasticity-oriented” treatment modality, requires further investigation.

Excitingly, there are a number of protocols for randomised controlled trials where ketamine will be compared against KAP in a TRD population. One will test ketamine with Almond therapy against ketamine with TAU (Chu et al. 2023), whilst another will examine the success of ketamine and BA against ketamine alone (Beaglehole et al. 2024). Such trials are necessary to test the true effectiveness of KAP, and the author eagerly awaits publication of these results.

## Conclusions

To conclude, current treatment options remain limited, applying more specifically to TRD than MDD. It is estimated that 1 in 3 people develop a treatment resistant form of the disorder. Ketamine is an effective treatment option, but the long-term effects are not fully known, and there is a need to discover ways to sustain or improve its AD effects.

KAP is a novel treatment option; it can be effective in alleviating depressive symptoms with sustained results for up to 6 months. However, it is a treatment that is very much in its infancy. For the future success of KAP, there is a need for randomised trials with a larger and more diverse sample population, control groups, and comprehensive blinding procedures.

## Supplementary information

Below is the link to the electronic supplementary material.

Supplementary Material 1 (DOCX 596 KB)

## Data Availability

No datasets were generated or analysed during the current study.
